# Minilaparoscopic over conventional laparoscopic cholecystectomy and appendectomy: is it worth it? A case series and review of literature

**DOI:** 10.1093/jscr/rjac136

**Published:** 2022-04-04

**Authors:** Davina Perini, Alessio Giordano, Tommaso Guagni, Stefano Cantafio

**Affiliations:** General Surgery Unit, Nuovo Ospedale “S. Stefano”, Azienda ASL Toscana Centro, Prato, Italy; General Surgery Unit, Nuovo Ospedale “S. Stefano”, Azienda ASL Toscana Centro, Prato, Italy; General Surgery Unit, Nuovo Ospedale “S. Stefano”, Azienda ASL Toscana Centro, Prato, Italy; General Surgery Unit, Nuovo Ospedale “S. Stefano”, Azienda ASL Toscana Centro, Prato, Italy

## Abstract

Minilaparoscopic cholecystectomy was proposed with the aim to improve the cosmesis and reduce the impact on the abdominal wall. Our aim was to analyze the knowledge currently available on this topic with a review of literature and with our experience to suggest patient-centered approach over the use of minilaparoscopic cholecystectomies and appendectomies. From January 2021 to October 2021, we performed 21 minilaparoscopic cholecystectomies and 12 minilaparoscopic appendectomies. Within the established 1-month and 3-month follow-up intervals, clinical examination and scar evaluation were assessed and a satisfaction questionnaire was completed by all the patients. No intraoperative or postoperative complications were recorded. Patients’ pain decreases significantly during hospital stay and 30 patients (90,1%) were discharged with VAS 0. The same happened with aesthetic score, that was 2,23 the postoperative-day-1, decrease to 1,87 1 week later and was 1,12 at 1- and 3-month follow-up.

## INTRODUCTION

Conventional laparoscopic cholecystectomy (CLC) is the treatment of choice for symptomatic cholelithiasis. The first CLC was performed by Erich Muhe in 1984 [1], in order to reduce the invasiveness of open conventional approach. Since the early 90’s, minilaparoscopic cholecystectomy (MLC) was proposed with the aim to further improve the outcomes in terms of cosmesis and impact on the abdominal wall [2, 3].

Minilaparoscopic surgery is generally used to indicate a laparoscopic procedure with smaller incisions and/or fewer ports [2]. Previous reviews compared studies reporting laparoscopic procedures performed either with minilaparoscopic instruments or with few trocars. Some reviews on this specific topic have already been published in last years, but they do not give any clear conclusions on which is the best surgical approach due to the relative scarcity of randomized trials and some obvious selection bias [4–8].

**Figure 1:**
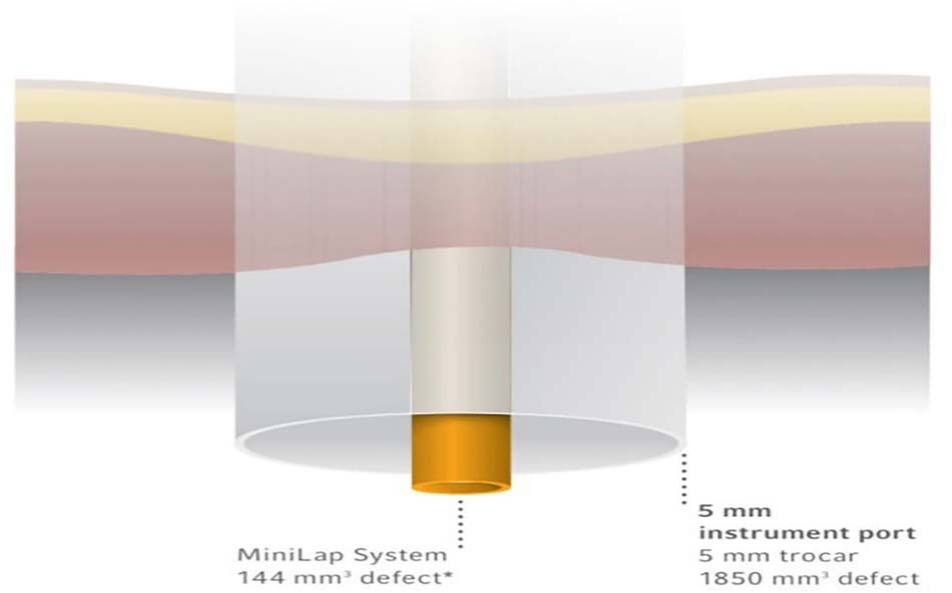
The MiniLap® Percutaneous Surgical System has a calculated defect volume that is more than 12 times smaller than an average 5-mm trocar.

The MiniLap System allows surgeons to insert the 2.4-1mm shaft diameter instrument percutaneously through the skin using an integrated needle tip with the goal of smaller incisions and less tissue trauma without any change in surgical technique (Fig. 1).

We choose to apply minilaparoscopic approach not only to cholecystectomies, as reported in literature, but also to appendectomies, concerning whom the literature is nearly non-existent.

The aim of the present study is to shed light on the knowledge currently available on this topic and to suggest a patient-centered approach over the use of minilaparoscopic techniques.

## CASE SERIES

From January to October 2021, 21 patients with symptomatic cholelithiasis and 12 patients with appendicitis consented to undergo minilaparoscopic surgery with MiniLap System (Teleflex Incorporated, Morrisville, NC, USA) and the MiniGrip^®^ Handle (Teleflex Incorporated).

Female patients outweighed male with a ratio 22:11. Mean age was 35.7 years, and mean body mass index was 26 (18–30) kg/m^2^. The characteristics of the examined patients are summarized in the Table 1.

Entry into the abdomen was achieved under direct visualization, placing a 5-mm trocar at the umbilicus, then pneumoperitoneum was created. After insufflations, a 30° 5-mm camera was inserted, and an initial diagnostic laparoscopy was performed. Once explorative laparoscopy was done, we choose whether to perform conventional laparoscopic surgery or minilaparoscopic one.

### Minilaparoscopic cholecystectomy

After exploring abdominal cavity, a second 5-mm trocar was introduced in the left umbilical, 10 cm left and upper the umbilicus, a 5-mm trocar in the subxifoid area and a MiniGrip^®^ handle in the right epigastrium. The integrated needle tip allowed direct insertion through the skin so that a 5-mm trocar was eliminated. The next steps are the same as the CLC. The gallbladder was retrieved through the umbilical port. The fascia was closed with 0-vycril. Subcuticular suturing was used for the incisions of the trocars, while the percutaneous access site was closed with simple stiches. Patients were discharged after meeting standard criteria, including adequate pain control management and resumption of oral intake. All of them were followed up 1 month and 3 months postoperatively.

**Table 1:** General characteristics of patients

	Minilaparoscopic cholecystectomies (*n* = 21)	Minilaparoscopic appendectomies (*n* = 12)	Overall (*n* = 33)
Mean age (years)	42.1	23.6	35.7
Gender (F:M)	14:7	8:4	22:11
BMI (Kg/m^2^)	27 (19–30)	24 (18–27)	26 (18–30)
ASA score	I (*n* = 5); II (*n* = 2)	I (*n* = 6)	I (*n* = 11)–II (*n* = 2)
Operative time (minutes)	55 ± 15	45 ± 10	50 ± 12

All patients that underwent cholecystectomy were diagnosed as cholelithiasis, none experienced cholecystitis or any other signs of inflammation. Patients were preoperatively assessed and classified as low-risk patients (American Society of Anesthesiologists I and II).

### Minilaparoscopic appendectomy

After exploring abdominal cavity, a second 5-mm trocar was introduced in the left iliac area and a MiniGrip^®^ handle in the right iliac. The next steps are the same as the conventional appendectomy. The appendix was retrieved through the umbilical port. Patients were discharged after meeting standard criteria, including adequate pain control and flatus. All of them were followed up 1 month and 3 months postoperatively. All patients that underwent appendectomy were diagnosed as appendicitis without peritoneal abscess or peritonitis. Patients were preoperatively assessed and classified as low-risk patients (American Society of Anesthesiologists I and II).

As far as the operation was concerned, mean surgical time was 55 minutes for cholecystectomies and 45 minutes for appendectomies. No patient required conversion to an open procedure, and there were no intraoperative complications. Drainages were not necessary. Within 24 hours postoperatively, patients were discharged from hospital. No cases of postoperative complication due to surgical technique were marked.

### Cosmetic results and patients’ satisfaction

**Figure 2:**
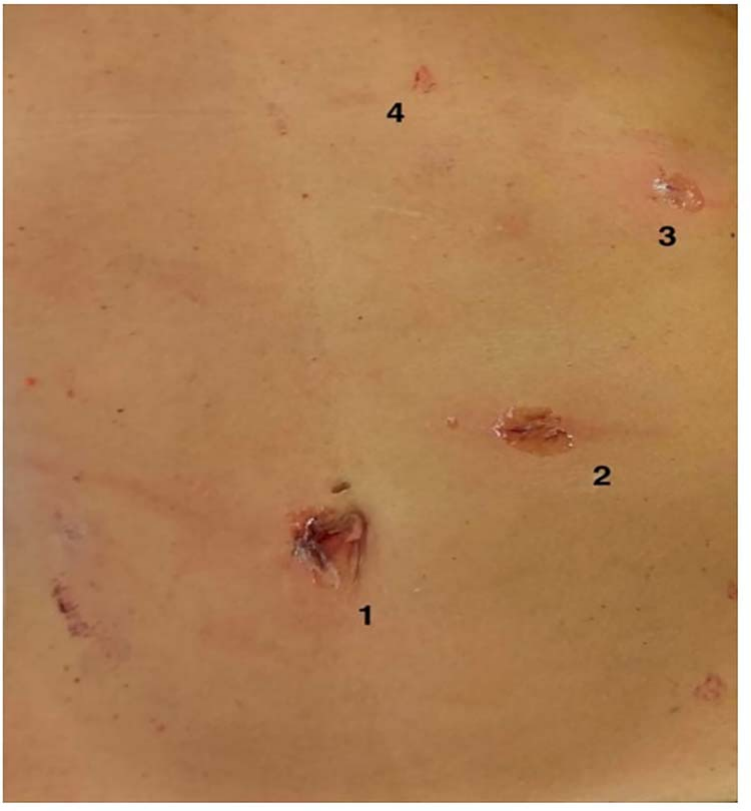
Trocars’ layout in minilaparoscopic cholecystectomy. (1) optic trocar, (2) 10-mm trocar, (3) 5-mm trocar and (4) Minigrip.

In 28 of the 33 patients (84,8%), the percutaneous access site was almost invisible even the first postoperative day and 1 week later (Fig. 2).

Within the established 1-month and 3-month follow-up intervals, clinical examination and scar evaluation were assessed and a satisfaction questionnaire was completed by all the patients.

Patients’ postoperative pain was scored with visual analog scale (VAS) between 0 and 10. Aesthetic score was evaluated with a score from 1 (scars almost invisible) to 4 (wound infection/complication).

Patients’ pain decreases significantly during hospital stay and 30 patients (90,1%) were discharged with VAS 0 (only two patients were discharged with VAS 1–2, but it was 0 a week after). The same happened with aesthetic score, that was 2,23 the postoperative-day-1, decrease to 1,87 1 week later and was 1,12 at 1- and 3-month follow-up.

## DISCUSSION

Some reviews about the comparison between MLC and CLC have already been published; however, in the last years, further studies have been made available, hence we decided to analyze this topic, including our experience, with the attempt to reach a clear conclusion on which is the best approach. (Table 2) For the review of literature, 5 electronic databases (EMBASE, MEDLINE, Pubmed, The Cochrane Central Register of Controlled Trials and Web of Science) were searched to identify titles and abstracts of all possible randomized control trials relevant to the topic of interest. All databases were searched from 1991 to 2021. The following terms were used to find eligible trials: ‘needlescopic’ or ‘miniport’ or ‘minilaparoscopic’ or ‘microlaparoscopic’, ‘cholecystectomy’, ‘appendicectomy’.

**Table 2:** Minilaparoscopic cholecystectomy: review of literature

Author and year	Comparison	No. of patient (total)	No. of patient (MLC)	No. of patients (CLC)	Complications	Operative time (min, median)	Conversion rate	Miniport size
Bisgaard *et al.* (2002) [4]	MLC vs CLC	52	25	27	–	65 vs 62	–	3.5
Sarli *et al.* (2003) [9]	MLC vs CLC	135	67	68	13 vs 12 (any complications)	50 vs 45	3 vs 4 (5.2% in total)	3
Huang *et al.* (2003) [11]	MLC vs CLC	49	24	25	6 vs 3	64.8 vs 47.3	5 (16.7%) from MLC to LC	2
De Carvalho *et al.* (2013) [12]	MLC vs CLC	41	18	23	–	45 vs 42	3 (17%) from MLC to LC	2.8
Alhashemi *et al.* (2017) [13]	MLC vs CLC	75	33	42	1 vs 1 (intraop.); 2 vs 1 (postop.)	73 vs 67	17 vs 1	3
Saad *et al.* (2013) [14]	SLC vs MLC vs CLC	70	35	35	8 vs 0 vs 0	45.7 vs 47.3 vs 35.0	–	3

In 2011, Thakur [5] broadly examined the differences between MLC and CLC on a variety of patient important outcomes, such as failure of surgical technique, adverse events related to surgery and cosmesis. They demonstrate that novel MLC are superior to CLC in terms of cosmesis and adverse events. However, minilaparoscopy were more likely to require a transition to conventional laparoscopy or open surgery.

The most recent systematic review and meta-analysis was published in 2020 by Coletta *et al.* [6] They analyzed fifteen studies and they concluded that both surgical approaches resulted substantially equivalent to perform CLC, with some advantages of conventional approach as for operative time and of minilaparoscopic on concerning postoperative pain. However, in literature there is a wide difference between the results of different studies. The authors stated that it is not possible to suggest one approach rather than the other. CLC resulted faster than MLC that showed to be less painful. Both surgical approaches resulted similar in overall complications and cosmetic results.

Results concerning these tasks of previous published reviews are discordant. The worth of Coletta review is to have partially avoided some selection bias. In fact, in this meta-analysis, only studies reporting surgical procedures performed with four trocars and in elective setting were included with the aim to obtain homogeneous data. [6]

However, the main limitation of the previous published studies is the lack of standard scales to measure postoperative pain and cosmetic results. In addition, most of them are not randomized trials, so that selection bias are unavoidable [8].

Costs are beyond the scope of the present study and a cost–benefit analysis was not included in this trial, but it is safe to assume equipment costs are comparable to the conventional laparoscopic ones, as underlined by Sarli *et al.* [9].

As marked by Bisgaard *et al.* [10], it is noteworthy that using minilaparoscopic equipment proved feasible and that the costs associated with those instruments are comparable to the costs of the standard equipment for laparoscopic cholecystectomy.

They even found the surgical equipment used for MLC was technically satisfactory even in cases with gross anatomic findings of chronic cholecystitis/dense adhesions and that smaller trocars reduced incisional pain in the first postoperative week and improved the cosmetic results [10]. In our experience, it was not so. As noted by Huang *et al.* [11], if the gallbladder had become inflamed or if severe adhesion by the surrounding organs was noted, the jaws of the 2-mm grasp were too small to perform optimal traction, and the 2-mm dissector was insufficiently strong to manipulate and dissect the dense fibrous tissue. The small caliber of the instruments used were, ultimately, still too weak to dissect any dense fibrotic tissue encountered, or too small to effectively support and hold the inflamed and thickened wall of the gallbladder.

We did not use a postoperative abdominal drain routinely, like De Carvalho *et al.* [12] did. The use of a drain will have an influence in the postoperative pain, causing more discomfort and therefore probably leading to higher pain scores.

Results concerning OT of previous published reviews are discordant, Hosono and Osaka [7] and Thakur *et al.* [5] reported shorter OT in the case of CLC and conversely McCloy *et al.* [8] reported in the case of minilaparoscopic one. Our experience has shown not significant differences in the operative time of the two different approaches.

As expected, considering the comparisons of the two approaches, no significant difference was observed in terms of overall morbidity, intraoperative bleeding, gallbladder perforation, and bile duct and bowel injury, also when individually analyzed [6–8, 11, 12].

Regarding this outcome, the studies present in literature are in contrast: earlier reviews reported better cosmesis for patient undergone minilaparoscopy, while recently conventional laparoscopy shows superiority [5, 7–9, 11–13].

In our study, both surgical techniques had optimum aesthetic results, with minilaparoscopy that had more satisfying results in shorter time. It is obvious that the lack of standardization in cosmetic evaluation affects the results.

Saad *et al.* compared MLC versus single-port (SP) versus CLC. No difference was apparent after 1 year, indicating that the improved cosmetic result achieved with SP and MLC techniques is only a short-term effect [14].

They clearly support a cosmetic advantage of SP, but at the expense of increased operating time and without a clear reduction in postoperative pain. Even the cosmetic advantage of SP was evident only in the short term in this study and not at 1-year follow-up [14].

Regarding the secondary endpoints, as for postoperative hematomas and incisional hernias, we did not observed any of this postoperative complications.

According to Thakur [5], the measurement of the pain scores and use of postoperative analgesia are not as important in the assessment of MLC as the evaluation of other key outcomes such as conversion to CLC or open cholecystectomy, which indicate failure of technique, or the occurrence of adverse events such as biliary injury [12].

Alhashemi *et al.* [13] particularly focused on an aspect that we did not consider: the return to normal physical activity and they found out that the recovery of physical activity was similar after MLC and CLC. MLC resulted in less fatigue and better scar appearance and satisfaction.

These wide differences in primary and secondary outcomes among different studies are probably due to the different meaning adopted to define MLC, including the reduced trocar surgeries. Furthermore, in the previous reviews, emergency procedures were included in the analysis.

Thus, it is necessary to consider only studies reporting surgical procedures performed with four trocars and in elective settings, with the aim to obtain homogeneous data. In fact, we enrolled patients with uncomplicated gallbladder diseases requiring laparoscopic cholecystectomy. Patient with previous acute cholecystitis, morbid obesity or previous upper abdominal surgeries were excluded, eliminating a subgroup of cases that are more technically challenging. Indeed, we first explored the abdominal cavity, then we choose which approach to adopt (minilaparoscopy in the case of clear abdominal cavity and no or negligible signs of inflammation, conventional laparoscopy in the other cases). Furthermore, the lack of standard scales to measure postoperative pain and, above all, cosmetic outcomes could lead to wrongful conclusions in comparison with the two surgical approaches.

Therefore, the minilaparoscopic cholecystectomies and appendectomies are safe and feasible procedures in highly selected patients, with negligible adverse events and conversion rate and excellent results in terms of cosmesis. Further truly randomized trials are needed to determine whether minilaparoscopic approaches truly offer any advantages.

### STATEMENT

Authors state that the work described has not been published previously, which it is not under consideration for publication elsewhere and that its publication is approved by all authors.

Conflict of interest statement. Authors certify that there is no actual or potential conflict of interest in relation to this article and they state that there are no financial interests or connections, direct or indirect or other situations that might raise the question of bias in the work reported or the conclusions, implications or opinions stated—including pertinent commercial or other sources of funding for the individual author(s) or for the associated department(s) or organization(s), personal relationships or direct academic competition.

## AUTHORS’ CONTRIBUTIONS

All authors contributed equally to this work.

## FUNDING

The authors state that no funding has been received for this article**.**

## References

[1] Mühe E. Laparoscopic Cholecystectomy—Late Results. Langenbecks Arch Chir Suppl Kongressbd 1991;1991:416–23. 10.1007/978-3-642-95662-1_189 1838946

[2] Dan AG, Mirhaidari S, Pozsgay M, Standerwick A, Bohon A, Zografakis JG. Two-trocar cholecystectomy by strategic laparoscopy for improved cosmesis (SLIC). J Soc Laparoendoscopic Surg 2013;17:578–84. 10.4293/108680813X13693422520242 PMC3866062 24398200

[3] Ahmad A, Arellano JJ, Agarwala A, Ahmad Z, Ahmad Z. A percutaneous technique of liver retraction in laparoscopic bariatric & upper abdominal surgery. Surg Obes Relat Dis 2016;12:1626–9. 27639986 10.1016/j.soard.2016.08.014

[4] Bisgaard T, Klarskov B, Trap R, Kehlet H, Rosenberg J. Pain after microlaparoscopic cholecystectomy: a randomized double-blind controlled study. Surg Endosc 2000;14:340–4. 10790551 10.1007/s004640020014

[5] Thakur V, Schlachta CM, Jayaraman S. Minilaparoscopic versus conventional laparoscopic cholecystectomy: a systematic review and meta-analysis. Ann Surg 2011;253:244–58. 21183848 10.1097/SLA.0b013e318207bf52

[6] Coletta D, Mascioli F, Balla A, Guerra F, Ossola P. Minilaparoscopic cholecystectomy versus conventional laparoscopic cholecystectomy: an endless debate. J Laparoendoscopic AdvSurg Tech 2021;31:648–56. 10.1089/lap.2020.0416 32833590

[7] Hosono S, Osaka H. Minilaparoscopic versus conventional laparoscopic cholecystectomy: a meta-analysis of randomized controlled trials. J Laparoendoscopic Adv Surg Tech 2007;17:191–9. 10.1089/lap.2006.0051 17484646

[8] McCloy R, Randall D, Schug SA, Kehlet H, Simanski C, Bonnet F, et al. Is smaller necessarily better? A systematic review comparing the effects of minilaparoscopic and conventional laparoscopic cholecystectomy on patient outcomes. Surg Endosc 2008;22:2541–53. 18810546 10.1007/s00464-008-0055-1

[9] Sarli L, Iusco D, Gobbi S, Porrini C, Ferro M, Roncoroni L. Randomized clinical trial of laparoscopic cholecystectomy performed with mini-instruments. Br J Surg 2003;90:1345–8. 14598412 10.1002/bjs.4315

[10] Bisgaard T, Klarskov B, Trap R, Kehlet H, Rosenberg J. Microlaparoscopic vs conventional laparoscopic cholecystectomy. Surg Endosc Other Interv Tech 2002;16:458–64. 10.1007/s00464-001-9026-5 11928028

[11] Huang MT . Minilaparoscopic and laparoscopic cholecystectomy. Arch Surg 2003;138:1017. 12963662 10.1001/archsurg.138.9.1017

[12] de Carvalho LFA, Fierens K, Kint M. Mini-laparoscopic versus conventional laparoscopic cholecystectomy: a randomized controlled trial. J Laparoendoscopic Adv Surg Tech 2013;23:109–16. 10.1089/lap.2012.0349 23276249

[13] Alhashemi M, Almahroos M, Fiore JF, Kaneva P, Gutierrez JM, Neville A, et al. Impact of miniport laparoscopic cholecystectomy versus standard port laparoscopic cholecystectomy on recovery of physical activity: a randomized trial. Surg Endosc 2017;31:2299–309. 27655375 10.1007/s00464-016-5232-z

[14] Saad S, Strassel V, Sauerland S. Randomized clinical trial of single-port, minilaparoscopic and conventional laparoscopic cholecystectomy. Br J Surg 2013;100:339–49. 23188563 10.1002/bjs.9003

